# Genome-wide screens in yeast models towards understanding chronological lifespan regulation

**DOI:** 10.1093/bfgp/elab011

**Published:** 2021-03-17

**Authors:** Luc Legon, Charalampos Rallis

**Affiliations:** School of Life Sciences, University of Essex, Wivenhoe Park, Colchester CO4 3SQ, UK; School of Life Sciences, University of Essex, Wivenhoe Park, Colchester CO4 3SQ, UK

**Keywords:** ageing, budding yeast, fission yeast, chronological, phenomics, TOR

## Abstract

Cellular models such as yeasts are a driving force in biogerontology studies. Their simpler genome, short lifespans and vast genetic and genomics resources make them ideal to characterise pro-ageing and anti-ageing genes and signalling pathways. Over the last three decades, yeasts have contributed to the understanding of fundamental aspects of lifespan regulation including the roles of nutrient response, global protein translation rates and quality, DNA damage, oxidative stress, mitochondrial function and dysfunction as well as autophagy. In this short review, we focus on approaches used for competitive and non-competitive cell-based screens using the budding yeast *Saccharomyces cerevisiae*, and the fission yeast *Schizosaccharomyces pombe*, for deciphering the molecular mechanisms underlying chronological ageing. Automation accompanied with appropriate computational tools allowed manipulation of hundreds of thousands of colonies, generation, processing and analysis of genome-wide lifespan data. Together with barcoding and modern mutagenesis technologies, these approaches have allowed to take decisive steps towards a global, comprehensive view of cellular ageing.

## Introduction

**Figure 1:**
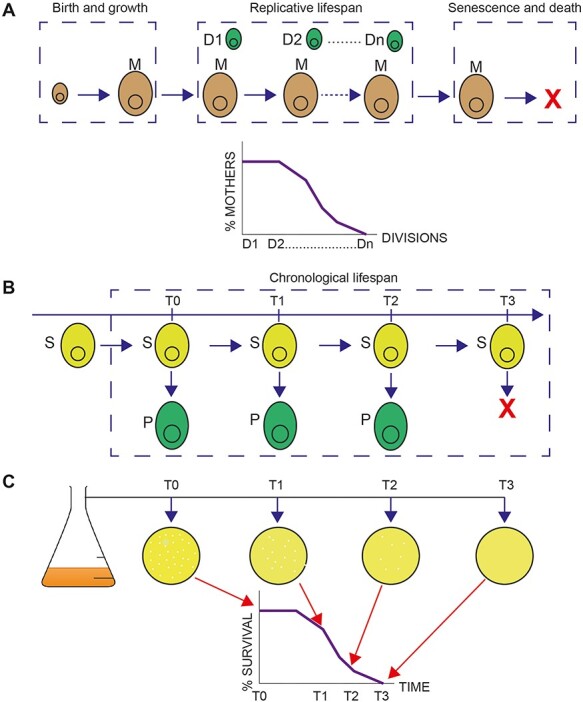
Cellular lifespan models. **A**. Replicative lifespan is defined as the number of mitotic divisions that a given mother cell can undergo until it reaches senescence and death. The schematic follows the life history of a ‘mother’ budding yeast cell (M) from its birth. Mitotic divisions and daughter cells (D1, D2, Dn) are numbered and recorded manually or using microfluidics, until senescence stage and death (marked as X). The process is performed for several mother cells. The overall data are used to generate a replicative lifespan curve. **B**. Chronological lifespan is defined as the time a postmitotic cell population (S, stationary phase culture) can give rise to progeny (P, able to revive). The schematic shows budding yeast cells. At specific timepoints, a sample is taken, and cells are plated in rich media allowing for re-entry to the cell cycle. The sampling and plating are repeated until the population can no longer revive (marked with X). This last timepoint defines the maximal chronological lifespan of the population. Alternatively, vital dyes such as Phloxin B are utilised for assaying metabolically active cells (see main text for details). **C**. Principle of CFU approach for determination of chronological lifespan. Cultures of strains to be characterised are grown to stationary phase. Colony numbers are counted and compared with 100% viability at the beginning of the assay and used to generate a chronological lifespan curve.

Two major cellular lifespan paradigms are currently utilised in the field of biogerontology: replicative (RLS) and chronological lifespan (CLS). RLS is the number of cell divisions that a mother cell can undergo until it reaches senescence and death (Figure 1A). CLS is defined as the time that a postmitotic population is viable [1] (Figure 1B). While the first is measured by counting (manually [2] or through microfluidics [2]) the number of daughter cells that arise from single cells, the latter is monitored with two ways: firstly, through clonogenic assays and measuring the number of colony forming units (CFU) generated with the passage of time [1, 3–5]. In this case, the proportion of cells able to divide and give rise to progeny is measured (revivability approach). During these assays, the proportion of cells able to divide is normalised to the measurement of initial timepoint that corresponds to the 100% viability. Secondly, viable cells can be assayed through staining with vital dyes such as Brilliant white (Phloxin B) and either microscopic/colony colour scoring or flow cytometry measurements [6–11]. The absolute viability measurements obtained for a given population using the two aforementioned approaches may differ: this is because a proportion of cells not able to revive, can still exhibit biochemical, signalling or metabolic activities and are still structurally intact [3, 10]. However, mutants that are scored long- or short-lived with the clonogenic assay are most likely to show the same lifespan phenotype with the vital stain [3, 9, 10].

Organismal ageing depends on both types of cellular lifespan. Ageing hallmarks and age-related diseases can be related to depletion or pathological physiology of either dividing adult stem cell pools or differentiated postmitotic cell populations [12]. In other words, organismal ageing has a cellular component that can be analysed in multiple levels (such as the levels of metabolome [11], transcription [13, 14], protein translation and misfolding [15], cellular architecture and cytoskeleton [16]), with appropriate cellular models. Single-celled organisms such as the budding (*Saccharomyces cerevisiae*) and the fission (*Schizosaccharomyces pombe*) yeasts have been pivotal in this aspect providing molecular insights and having huge conceptual contributions in the field. Characterising the contribution of individual mutants in ageing is a continuously active and informative activity in the field. On top of these studies, genome-wide screens have provided insights on the role of evolutionarily conserved processes and signalling pathways in ageing such as nutrient response [17, 18], protein translation, oxidative damage [19, 20], mitochondrial function [21, 22] and autophagy [22, 23] opening new avenues for biogerontology research. Yeasts have proved informative and helped in understanding mechanisms of highly conserved pathways (from yeast to human) in physiology, health and disease such as the Target of Rapamycin (TOR) [24], glucose sensing (PKA) and stress response pathways (Sty1/p38) [25].

A pivotal step for conducting genome-wide lifespan screens has been the availability of deletion libraries for both yeasts [26–28]. Conducting ageing screens by massive parallel lifespan measurement of thousands of mutants, has been the first step. Such approaches where mutants are grown and assessed separately are known as noncompetitive screens and have been extremely fruitful. Barcoding of the library mutants and development of relevant sequencing technologies, together with various mutagenesis platforms [29–34] or inter-crossing approaches [35] have led to screens where thousands of mutants are grown in a pool and their chronological lifespans are assessed based on the abundance of barcodes. These are known as competitive assays and have complemented non-competitive approaches providing novel insights. Here, we showcase some of the approaches utilised for competitive and non-competitive screens that have contributed to a better understanding of ageing mechanisms and provide a few examples of the generated outcomes. High-throughput developments on the wet-lab are accompanied with new computational tools that provide quality control steps, faithful quantitation of the lifespan trait with appropriate statistics and user-friendly graphical representation of the obtained results.

## Non-competitive screens for factors affecting chronological lifespan

**Figure 2:**
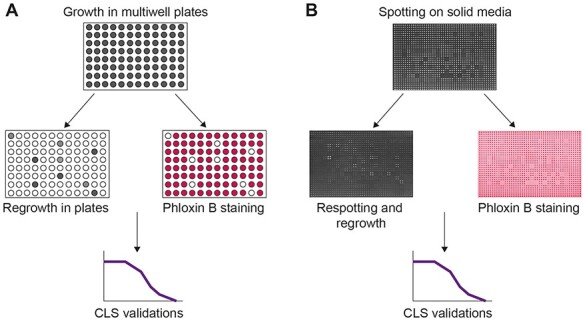
Non-competitive high-throughput lifespan screens that have been used for fission yeast. **A**. A liquid culture setup where strains are individually grown in multi-well plates. Their viability is assessed either through regrowth in new plates (where growth kinetics are used as a proxy for the proportion of live cells in the initial wells) or through staining with vital dyes such as phloxin B. **B**. A solid media spotting setup for genome-wide ageing screens. Strains are spotted, grown, and aged in colonies. Viability is assessed either through re-spotting using colony growth as viability/fitness proxy or through staining with phloxin B. In all cases, mutants/strains of interest are validated with traditional lifespan assays using the CFU approach (shown in Figure 1C).

Following a multitude of environmental, nutritional and drug screens, yeast deletion libraries have been used for lifespan studies, with the budding yeast system pioneering within the field. A first step using mutant library resources has been the contribution of all non-essential genes in lifespan regulation. For his reason, a genome-wide study of genetic factors affecting chronological lifespan was devised in budding yeast [18] using small volume liquid cultures (see Figure 2A). Cultures were initially grown in 96-well plates until reaching stationary phase. At various intervals, a sample from each well is transferred into a corresponding well of a second plate. The optical density (OD) at 600 nm of each well was determined using a plate-reader. The OD corresponded to the number of viable cells and the approach was validated with CFU assays. The authors screened 4800 mutants and 16 of the longest-lived mutants were related to TOR signalling and nutrient sensing. Some TOR-implicated and TOR-regulating genes (SLM4, GTR1, GTR2, MEH1 and NVJ1) have been implicated in the regulation of autophagy [35–39]. Since TOR had been implicated in replicative lifespan, the authors with subsequent experiments showed that it also controls chronological lifespan in budding yeast. The aforementioned study has been one of the best examples towards identifying a highly conserved, nutrient response and proageing signalling pathway.

Acceleration and efficiency of relevant screens was achieved through automation and in combination with relevant pharmacological interventions targeting specific ageing pathways. Robotics have revolutionised cell-based screens addressing questions on toxicity, stressor and drug sensitivity or resistance as well as genome-wide genetic interactions in the presence and absence of drug treatments revealing insightful positive and negative gene relationships. Such approaches are conducted through spotting colonies on solid media (Figure 2B). Relevant experiments in fission yeast showed that TOR is implicated in the chronological lifespan of this model organism too [5]. Subsequent genome-wide screen for resistance to TOR inhibition through a combination of caffeine and rapamycin, involving around 3000 deletion mutants, revealed new players in chronological ageing and uncovered a relationship between cellular growth and lifespan [3]. In a relevant Torin1-resistance screen (Torin1 is an ATP-competitive inhibitor of the TOR pathway), Gaf1 a GATA transcription factor [40, 41] was shown to be required for normal chronological lifespan in fission yeast. In addition, Gaf1 was partially required for Torin1-dependent lifespan extension and defined a novel mechanism of transcriptional control of protein translation [4]. Such studies open up opportunities to assess whether novel mechanisms in physiology, ageing and disease, also apply in metazoa.

Beyond characterisation of short and long-lived mutants a quest in the field of biogerontology is to identify compounds that can increase cellular fitness and lifespan. Indeed, chemical genetic screens using yeasts have been very fruitful. A chemical genetic screen for compounds that extend the chronological lifespan of fission yeast led to the discovery of eight natural products with such properties [42]. Further experiments uncovered antiageing pathways. The ionophores monensin and nigericin extended lifespan through vacuolar acidification. The effect depended on the vacuolar ATPase (V-ATPase) subunits Vma1 [43] and Vma3 [44]. Prostaglandin J₂ behaved as an antiageing compound through inhibiting mitochondrial fission. Prostaglandin J₂ lifespan-extending effects depend on the mitochondrial fission protein Dnm1 and the G-protein-coupled glucose receptor Git3. The latter functions within the pka1 signalling pathway, known from separate studies to be largely implicated in lifespan regulation [10, 11, 45]. Additionally, mycophenolic acid (MPA) and acivicin, two chemicals that inhibit guanosine monophosphate (GMP) synthesis, extended chronological lifespan, indicating that an imbalance in guanine nucleotide levels impinges upon longevity. Diindolylmethane (DIM), tschimganine and the compound mixture mangosteen extended survival of post-mitotic populations. This work is a great paradigm of phytochemicals that can be beneficial in lifespan and further studies will be required to fully elucidate their effects in multicellular systems and human cells towards translational drug development. Further chemical genetic screens affecting mitochondrial function and electron transport chain have revealed the role of reactive oxygen species, intact respiratory function and retrograde signalling in cellular growth and chronological ageing [21, 46]. The mentioned examples showcase the power of yeast cell-based screens in toxicity testing, fitness as well as in ageing studies.

Two models of chronological lifespan have been developed in fission yeast. During the glucose depletion (or stationary phase) model, cells are arrested in G2 or a mixture of G1 and G2 depending on the media used (YES or EMM2, respectively), and can survive in this state for several days [5, 9, 10, 45]. A second model uses depletion of nitrogen (but an abundance of glucose) where the cells undergo two divisions, and arrest in G1/G0 phase of the cell cycle [47–49]. The cells can survive in a G0 quiescent state for several months, while being metabolically active, especially for processes such as autophagy and proteasome-mediated protein degradation [22]. The developed models have been used in various conditions with some of them showing molecular signatures that simulate caloric restriction. For example the cells in the stationary model can be longer-lived when grown in 0.5% instead of 3% glucose [9, 10]. Genome-wide screens have been conducted using both models providing information on genes and pathways implicated in chronological lifespan as well as quiescence entry and maintenance. For example, a screen of 610 temperature-sensitive mutants has identified 33 genes that are required for entry into and maintenance of quiescence. These genes encode for proteins that are involved in stress-responsive and cell-cycle kinase signalling pathways, actin-bound and osmo-controlling endosome formation, RNA transcription, splicing and ribosome biogenesis, chromatin silencing, biosynthesis of lipids and ATP, cell-wall and membrane morphogenesis and protein trafficking and vesicle fusion [48]. Fcp1, a CTD phosphatase of RNA polymerase II, differentially affects the gene transcription was specifically highlighted as being pivotal in differentiating quiescence from proliferation [48]. In a relevant comprehensive G0 maintenance screen [49] of the fission yeast deletion library [28, 50], it was revealed that 85 genes are required to maintain mitotic competence during the G0 phase induced by nitrogen deprivation. Out of these genes, a significant number were phosphatase-related genes implying that dephosphorylation is a primary means of maintaining mitotic competence in G0. In addition, 7 autophagy genes and 13 genes related to genome structure were identified [49]. The use of the nitrogen starvation model for ageing has, therefore, proved powerful and complementary to the stationary model and revealed conserved factors that are important for maintenance to long-term quiescence.

Although the gene list of lifespan-controlling genes has been continuously growing due to multiple genome-wide genetic and chemical screens, the relationship of these genes with ageing in other model systems has not been fully addressed. Towards this issue, Burtner and colleagues [51] performed a functional genomic analysis of chronological longevity for 550 single-gene deletion strains in budding yeast. Notably, the study identified 33 previously unknown determinants of CLS. However, there was no significant enrichment for increased lifespan in corresponding *Caenorhabditis elegans* mutants. Moreover, although a trend towards an overlap with genes enhancing replicative lifespan was observed, it was not significant. This study also showed through a screen for reduced acidification of the culture medium that 11 out of 76 deletions strains exhibiting reduced culture medium acidification were chronologically long-lived. Beyond indicating that acidification of culture media is an important determinant of survival during chronological lifespan, this work is important for the field as it has suggested that it **is** of benefit to assay the yeast CLS under multiple media conditions [51] to specify exact conditions that would provide overlaps with organismal ageing.

An important aspect of genome-wide cell-based assays is the overlap of their results. In a study comparing three different ageing screens in budding yeast [52], among 3209 strains present in all three screens, only nine deletions strains were in common as long-lived strains and 13 for short-lived mutants. Pairwise overlap between screens was low too. Gene ontology enrichment for long-lived strains did not generate any significant results. Downstream analyses showed that auxotrophic requirements, ploidy status, extrinsic factors such as media composition and aeration, as well as interactions that may occur between them, have significant impact on CLS outcomes [52]. Future studies should account for these factors and indeed recent experiments include prototroph strains at least for the validation of the outcomes from genome-wide screens.

Efforts towards efficient determination of lifespan in a high-throughput manner without the need of CFU determination led to alternative approaches in budding yeast with the use of flow cytometry (and propidium iodide stain) [53]. The method was validated with known mutants that alter lifespan and correlated with the CFU approach [53]. Flow cytometry methods using propidium iodide and YO-PRO-1 that enter into necrotic (and apoptotic cells in the case of YO-PRO-1) but not into alive cells were utilised in another notable study that identified quantitative trait loci (QTLs) that control lifespan in various states such as calorie restriction, rapamycin treatment and nutrient-rich environment. This was conducted through highly recombined budding yeast populations [54]. The study identified major QTLs related to the cell wall glycoproteins FLO11 and HPF1. Beyond uncovering interesting biology linking increased oxygenation to altered methionine, lipid and purine metabolism that resulted to shortened lifespan [54], this paper highlighted the power of crossing natural yeast strains for linking genotype to phenotype.

In addition to the flow cytometry method as alternative to the CFU approach, fast colorimetric approaches suitable for high-throughput screens have been devised. Notably, one of these methods makes use of the reduction of the yellow tetrazolium compound MTT (3-(4,5-dimethylthiazol-2-yl)-2,5-diphenyltetrazolium bromide) to a purple-coloured formazan product with an absorption peak at 550 nm within live cells [55]. The method is shown to be reliable during caloric restriction conditions and verified with known long and short-lived mutants. Using this technique in high-throughput settings the effects of rapamycin, metformin, resveratrol and that of the polyphenol (−)-epigallocatechin gallate (EGCG) have been examined. This report is important showing that such approaches can be used as initial rapid screens of compounds in combination with a collection of mutant strains.

### Competitive screens for factors affecting chronological lifespan

**Figure 3:**
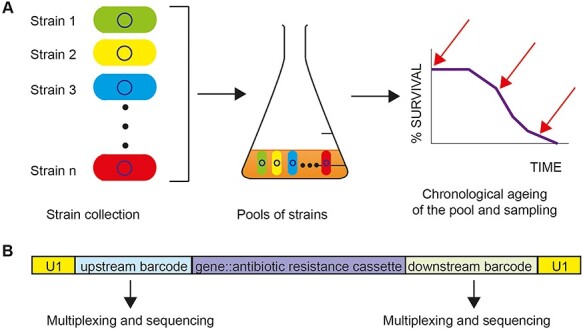
Barcode sequencing principle and use in lifespan screens. **A**. Thousands of barcoded strains are mixed, grown and aged in a pool. The viability of the pool is monitored with a classic CFU assay (Figure 1B) and samples are collected at specific intervals. **B**. Schematic of a locus for a deleted gene within each barcoded strain (example from fission yeast deletion library). Part or the whole ORF is substituted with an antibiotic resistance cassette which is flanked by unique upstream and downstream barcodes and universal sequences (U1 and U2). Barcodes from all collected timepoints of an ageing experiment are PCR amplified, multiplexed and sequenced. Based on barcode relative abundance long-lived and short-lived strains are revealed and validated by individual CFU (CLS) assays.

Contrary to non-competitive screens where individual mutants are separately grown either in liquid media or on solid media through manual or robot-mediated pinning (Figure 2, showing screens that have been used for fission yeast), during competitive screening mutants are mixed and grown together in pools (Figure 3A). Following ageing of the pool, the abundance of each mutant can be assessed through unique molecular tags or barcodes using microarray platforms [56] or barcode sequencing (Bar-seq) [57] (Figure 3B). The relative abundance of the barcodes at each timepoint are normalised with the reads obtained at time zero of the assay. Slow mutants can be filtered out either by using relevant information from growth assays of the pool [23] or via regrowing the aged sample for a set period and then isolate DNA for barcode sequencing [35]. The competitive and non-competitive approaches are complementary and can reveal non-overlapping information [23]. For example, a competitive screen can reveal mutants that are able to efficiently uptake and assimilate nutrients from their environment (from dead cells). In this example, such mutants can potentially perform well in a competitive screen as opposed to a non-competitive one. Nevertheless, a competitive screen bypasses the handling of tens or hundreds of tubes and plates with the contamination dangers especially for months-long ageing screens.

A competitive ageing assay was performed in budding yeast where samples from the ageing pool were collected at specific timepoints [58]. Mutants were then detected using a microarray DNA hybridization technique that quantifies abundance of the barcode tags of each mutant. Using this approach multiple short- and long-lived mutants were identified with autophagy mutants being among the short-lived and mutants coding for proteins involved in *de novo* purine biosynthesis pathway, which ultimately produces IMP and AMP were among the long-lived ones [58]. Validation experiments targeting autophagy or purine biosynthesis has the expected lifespan outcomes. In a similar approach, deletion of genes involved in protein sorting in vacuoles, autophagy and mitochondrial function shortened life span, confirming that respiration and degradation processes are essential for long-term survival. Among the genes whose deletion significantly extended life span were genes implicated in fatty acid transport and biosynthesis, cell signalling and transfer RNA (tRNA) methylation such as ACB1, CKA2 and TRM9, respectively [59].

An interesting screen pipeline was proposed in budding yeast that utilised fluorescently labelled deletion strains (e.g. with RFP) and wild-type/control (e.g. with CFP) [60, 61]. Deletion strains and control are separately grown to saturation and then mixed in a pairwise manner (control with each of the deletion strains) and at certain ratio to achieve a desired dynamic range. These competitive cultures are sampled each day and regrown while monitoring CFP, RFP signals and cell numbers. Using this approach chronological lifespan were determined even under dietary restriction revealing roles for Swr1 histone exchange complex and links between autophagy and lipid metabolism [60]. The aforementioned method was expanded by the development of data analysis approach that uses the change in fluorescent-signal ratio in the regrowing cultures to estimate the relative survivorship of each mutant [61]. Data is fitted to a multiple linear-regression model that takes in account multiple parameters such as growth rate of the mutant and survivorship over to the wild-type. This is an important report that has been tested for lifespan performance as well as for gene/drug interactions (metformin in this case) [61]. The authors made further steps towards acquirement of a global view of cellular ageing through additional screens in two nitrogen regimens: one state where cells are provided with a rich nitrogen source (glutamine) and one that the nitrogen source is poor and therefore cells are restricted (γ-aminobutyric acid) [62]. The authors identified hundreds of mutants with altered lifespan under these conditions. Mutants with diminished lifespan are related to vesicular transport, autophagy, respiration and protein translation. Mutants with increased lifespan are related to protein degradation, mitochondrial membrane and stress response. This study enriched our understanding of lifespan regulation in dietary restriction related to nitrogen [62].

Beyond using existing and commercially available deletion collections, scientists have developed an insertion vector-based method of generating independent barcode-tagged fission yeast insertion mutant libraries. The approach, with the potential of generating viable mutations in both essential and non-essential genes [63] led to a collection of 10 000 fission yeast insertion mutants organised as six pools of mixed mutants.. Aging of the insertion mutant pools led to the identification of a long-lived mutant bearing an insertion mutation in the cyclin gene *clg1* [64]. Clg1 protein physically associates with the cyclin-dependent kinase Pef1. Deletion of Clg1 or Pef1 was found to extend lifespan with the Pef1-dependent mechanism of lifespan extension involving the downstream protein kinase Cek1 [64]. This study is a paradigm that alternative competitive approaches can result in novel anti-ageing and pro-ageing factors that are conserved among eukaryotes.

Chronological lifespan is a complex trait that exhibits great variation [65–67]. Most screens have utilised deletion libraries that include mutants of protein-coding genes. Ellis *et al*. [35], have used a different approach to identify natural genetic variants contributing to cellular ageing. Using two strains of fission yeast that differ in chronological lifespan, segregant pools were generated and subjected to advanced intercrossing over multiple generations to break up linkage groups. The intercrossed segregant pool was then chronologically aged and genome sequencing was performed at various timepoints to detect variants that are enriched as a function of age [35]. Τwo candidate variants within Chromosome II of the long-lived strain represented small insertions and deletions in the 5′-untranslated regions of *ppk31* (coding for a Rim15 orthologue, a conserved kinase controlling cell proliferation in response to nutrients [68]) and *SPBC409.08* (a predicted spermine transmembrane transporter [69]). Further experiments supported that Ppk31 and SPBC409.08 may function together to modulate lifespan, thus linking Rim15/Ppk31 with spermidine metabolism [35].

While most ageing pool experiments involve glucose starvation similar experiments have been conducted using fission yeast and nitrogen starvation. Parallel mutant phenotyping of a prototroph version of the haploid deletion library [28] using Bar-seq has been used to assay pooled haploid deletion mutants as they aged together during long-term quiescence [23]. These are particularly demanding experimental setups in which fission yeast cells can survive for months and need weekly glucose refeeding. Lifespan scores could be calculated for 1199 mutants. Autophagy mutants were under-represented or not detected underlying the role of this process in maintenance within long-term quiescence [22, 47–49]. Genes encoding membrane proteins were particularly prominent as pro-ageing factors.

### Developed computational tools for lifespan determination

The clonogenic, revivability CLS assays are based on CFU determination and can be time-consuming requiring several parallel cultures, serial dilutions and multiple plating repeats followed by colony counting. Biogerontologists using cellular model systems are continuously trying to devise new reliable and user-friendly methodologies and computational tools that will allow faithful CLS determination in a medium to high-throughput manner in a plethora of conditions. Here we present a few of these tools developed for yeast lifespan analysis.

Analyser (Analytical Algorithm for Yeast Survival Rates) [70] is a software that allows automation and analysis of cell survival data from aging yeast cultures. The system uses fluorescent cell counter data or microplate imaging. The system is demonstrated to be efficient when testing the lifespan-extending effects of caloric restriction [70]. This software is adaptable, cost-effective and easy-to-easy for toxicity and drug treatment assays.

Yeast Outgrowth Data Analyzer (YODA) [71], is an automated system towards analysis of yeast cell population survival. The system is based on growth kinetics measured by optical density over time. The YODA Analyser was initially designed for yeast CLS quantitation. However, it can be used to quantitatively measure growth rates and survival of yeast cells in response to a multitude of environmental conditions differing on temperature, nutritional status, chemical stressors and drugs. While YODA has been optimised for use with a Bioscreen C MBR shaker/incubator/plate reader, it can be adapted to any standard plate reader or spectrophotometer. Pilot screens and trials show that use of YODA can potentially reduce the effort and resources required to measure CLS and analyse the resulting data by at least 15-fold [71].

Pyphe is a package providing viability scores from phloxine B staining or colony growth curves [72]. The program processes images of spotting robot-arrayed colonies that are acquired with transilluminating flatbed scanners. Using Pyphe, the obtained viability scores from quantifying the redness of phloxine-stained colonies accurately reflects the fraction of live cells within colonies [72]. Thus, the platform can be used as an entry point for large-scale lifespan screens in varied conditions.

Throughout the manuscript we have referred to regrowth or outgrowth from ageing cultures and using growth parameters as a proxy for survival of the examined mutants [73]. Traditionally, ageing cultures were kept in separate tubes or flasks making the whole process laborious and not suitable for a high-throughput screen. Jung *et al.* [74] developed wet-lab protocols for determination of both growth rates and parallel lifespan of hundreds or thousands of mutants. Ageing cultures were kept at 96-well plates with the regrowth step performed at 384-well plates [74]. These protocols were accompanied by freely the available software tools GATHODE and CATHODE. Both tools make use of readings from standard plate readers making the method approachable and user-friendly [74].

## Conclusions and perspectives

High-throughput screens have now revealed many genes and signalling pathways implicated in yeast chronological lifespan. In addition, mutant libraries and saturation mutagenesis in combination with chemical compound screens have uncovered life-prolonging chemicals and gene-drug genetic interactions in ageing.

Many genes are having orthologues in animal models and their role in ageing is validated in further downstream analyses or separate studies. Pharmacological treatments in combination with genetic and genomic approaches such as global genetic interactions (for example, through Synthetic Genetic Arrays [75, 76]) can help towards building a functional connectome for chronological lifespan and reveal a comprehensive global view of cellular ageing. Following cues from the yeast screens, CRISPR/Cas9 mediated screens and assays for in mammalian cells [77] can directly provide insights of orthologues and gene‑drug relationships even for human cellular lifespan, opening avenues for targeted interventions that would prevent or ameliorate age-related diseases.

**Key Points**

- Genome-wide ageing screens using yeasts have identified anti-ageing and pro-ageing genetic factors and signalling pathways.
- Screens can be non-competitive where thousands of mutants are grown separately, or competitive where mutants are pooled, and their abundance is quantified using mutant-specific barcodes.
- Wet lab protocols as well as computational tools have been developed for faster lifespan assays, quantitation and appropriate statistics.

## Notes

**Luc Legon** is a PhD student at the University of East London and visiting PhD student at the School of Life Sciences, University of Essex working on the role of mTOR signalling pathway in neural differentiation and neurodegeneration.

**Charalampos Rallis** is a Lecturer in Cellular Ageing at the University of Essex. The Rallis lab investigates nutrient-response pathways and metabolism in lifespan regulation. Rallis supervises Legon’s PhD studies.
